# Cardiometabolic profile of women with a history of overt diabetes compared to gestational diabetes and normoglycemia in index pregnancy: Results from CHIP‐F study

**DOI:** 10.1111/1753-0407.13461

**Published:** 2023-08-30

**Authors:** Yashdeep Gupta, Alpesh Goyal, Samita Ambekar, Mani Kalaivani, Neerja Bhatla, Nikhil Tandon

**Affiliations:** ^1^ Department of Endocrinology and Metabolism All India Institute of Medical Sciences New Delhi India; ^2^ Department of Statistics All India Institute of Medical Sciences New Delhi India; ^3^ Department of Obstetrics and Gynaecology All India Institute of Medical Sciences New Delhi India

**Keywords:** diabetes in pregnancy, gestational diabetes mellitus, overt diabetes in pregnancy, postpartum, South Asia

## Abstract

**Purpose:**

We aimed to evaluate the prevalence of postpartum diabetes among women with a history of overt diabetes in pregnancy (ODiP) and compare with women having a history of gestational diabetes mellitus (GDM) and normoglycemia in pregnancy.

**Methods:**

We have an established longitudinal cohort of postpartum women with a history of hyperglycemia (preexisting diabetes [PED] [*n* = 101], ODiP [*n* = 92], GDM [*n* = 643]), and normoglycemia (*n* = 183) in pregnancy. For this study, we excluded women with PED and invited other eligible women in a fasting state for clinical and biochemical evaluation.

**Results:**

We evaluated 918 women with a mean (SD) age of 33.6 (5.0) years and at a median (interquartile range) postpartum interval of 31 (20–45) months. Diabetes was diagnosed in 65 (70.7%) women in ODiP compared to 99 (15.4%) in GDM (*p* < .001) and 4 (2.2%) in normoglycemia group (*p* < .001). In the ODiP group, the prevalence of diabetes was 47.4% among women tested in the first year postpartum, increasing to 86.8% among women tested at >3 years postpartum. Diabetes was more common when ODiP was diagnosed in the first (27/29, 93.1%) compared to the second trimester of pregnancy (35/57, 61.4%). The adjusted odds ratio for diabetes in ODiP was 14.82 (95% confidence interval, 8.49–25.87; *p* < .001; reference category: GDM).

**Conclusions:**

The prevalence of postpartum diabetes was significantly higher in women with ODiP compared to GDM. Nearly 50% of women with ODiP did not develop diabetes in the first year of follow‐up, especially when ODiP was diagnosed after the first trimester of pregnancy and on the basis of a 2‐h oral glucose tolerance test value. Such women are amenable to prevention strategies.

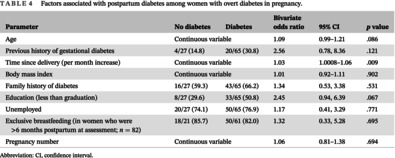

## Graphical abstract

Highlights

1. We evaluated the prevalence of postpartum diabetes among women with overt diabetes in pregnancy (ODiP).
2. Diabetes was diagnosed in 65 (70.7%) women with ODiP at a median postpartum interval of 29 months.
3. The prevalence of diabetes in the ODiP group was 47.4% among women tested in the first year postpartum, increasing to 86.8% among women tested at >3 years postpartum.
4. Postpartum diabetes was more common when ODiP was diagnosed in the first (27/29, 93.1%) trimester of pregnancy.
5. Postpartum diabetes was more common when ODiP was diagnosed based on glycated hemoglobin (HbA1c) elevation before 15 weeks of gestation (19/20, 95.0%).

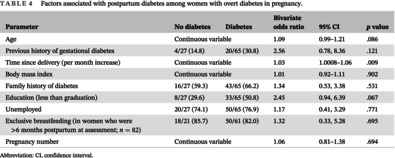

## 1 INTRODUCTION

In 2010, the International Association of Diabetes and Pregnancy Study Groups (IADPSG) proposed a new definition for gestational diabetes (GDM) and at the same time also introduced a new entity named “overt diabetes in pregnancy,” which represented a more severe form of hyperglycemia in pregnancy.^1^ The condition “overt diabetes in pregnancy (ODiP)” refers to hyperglycemia first diagnosed during pregnancy, which meets the definition of diabetes in a nonpregnant adult.^2^ ODiP constitutes nearly 9% of all cases of hyperglycemia in pregnancy and as per the International Diabetes Federation estimates, this condition affected around 1.9 million women in 2021.^3^ There is emerging evidence for increased incidence of adverse pregnancy outcomes among women with ODiP; however, data on long‐term glycemic and metabolic outcomes among such women are limited.^4^ The focus has more often remained on women with GDM in the postpartum period as this is the more common condition, with a greater awareness among practitioners.

With a steady increase in the rates of obesity and age at the time of conception,^5^, ^6^ the number of women with ODiP is likely to increase. This rise can impose enormous pressure on human and economic resources of the health care system. Globally, there are only three studies that have evaluated the prevalence of diabetes in women with ODiP in the first 6 months following childbirth.^7^, ^8^, ^9^ These include reports by Wong et al. (*n* = 133; 21% diabetes at 6–8 weeks postpartum),^7^ Park et al. (*n* = 71; 73% diabetes at 6–8 weeks postpartum),^8^ and Nabi et al. (*n* = 24; 25.0% diabetes at 6 months postpartum).^9^ Thus, long‐term glycemic data are limited and little is known about metabolic outcomes beyond 6 months postpartum in such women. We know that women with GDM have an increased cardiovascular risk and biologically, the association with cardiovascular diseases is likely to be stronger with ODiP.^10^ However, due to paucity of data, the current guidelines are often silent on the strategies for screening, management, and postpartum follow‐up of this high‐risk group; this limits our ability to improve the disease course in many such women.^11^, ^12^, ^13^

We have established a cohort of postpartum women with history of hyperglycemia and normoglycemia in pregnancy along with their offsprings and spouses for evaluating vertical and horizontal interaction of cardiometabolic risk factors among women–offspring and women–spouse dyads, respectively. In this first paper from our “***C**ohort Study of Indian Women with **H**yperglycemia **i**n **P**regnancy and their **F**amilies* (CHIP‐F)”, we report the long‐term prevalence of postpartum diabetes in women with ODiP and compare with women having a history of GDM and normoglycemia during their index pregnancy. We also report prevalence of other cardiometabolic risk factors such as hypertension, overweight/obesity, and metabolic syndrome among such women.

## 2 MATERIALS AND METHODS

### 2.1 Study design and settings

We performed this study at the All India Institute of Medical Sciences (AIIMS), New Delhi, India (a tertiary care center catering to patients predominantly belonging to low‐ to middle‐income groups) from 2019 to 2022. We collected data after obtaining approval from the ethics committee of our institute and written informed consent from each participating individual.

### 2.2 Study objectives

The primary objective was to evaluate prevalence of postpartum diabetes among women with ODiP. The secondary objectives were (a) to compare the prevalence of postpartum diabetes and other cardiometabolic risk factors between women with ODiP, GDM, and normoglycemia in index pregnancy; and (b) to evaluate factors associated with postpartum diabetes among women with ODiP.

### 2.3 Brief description of the cohort

At our institute, we have established a cohort of postpartum women (*n* = 1019) with history of hyperglycemia (preexisting type 1 or type 2 diabetes [*n* = 101], ODiP [*n* = 92], GDM [*n* = 643]), and normoglycemia (*n* = 183) during their index pregnancy. For this cohort, we approached all women with GDM, ODiP, and preexisting type 1 or type 2 diabetes who delivered between 2017 and 2021 and enrolled all those who provided written informed consent. In addition, we invited all women with GDM and ODiP who delivered in our hospital between 2012 and 2016 and participated in one of our earlier studies.^14^, ^15^ Given the large number of women with normoglycemia in pregnancy, we invited only those who participated in one of our earlier studies or those who were enrolled in the clinics of one of the investigators (YG) and delivered at AIIMS, New Delhi, during the 2016–2022 period.^15^, ^16^ Women were telephonically contacted, and those willing to participate were scheduled for the study visit. We made at least 3–5 attempts to contact women over a 4–6 weeks period. For the purpose of the current study, we excluded women with a history of preexisting diabetes and analyzed data of women with a history of ODiP, GDM, and normoglycemia in pregnancy.

We approached 2858 women, of whom 1019 agreed to participate in our overall cohort (participation rate: 35.7%). Given the lack of sufficient data on women who did not participate, we cannot report reasons for nonparticipation or whether these women differed from those who participated. However, we performed a sensitivity analysis on a limited number of variables in one of our earlier studies involving postpartum follow‐up of women with GDM.^14^ We found that the overall participation rate in our earlier study was 31.4% and women who participated were older and more likely to be on insulin during pregnancy than those who did not. Therefore, the participation bias, if any, due to differential characteristics of participants versus nonparticipants in this study cannot be ruled out.

### 2.4 Definitions

#### 2.4.1 Exposure

GDM and normoglycemia in pregnancy were defined per World Health Organization (WHO) 2013 criteria.^17^ The WHO criteria use IADPSG thresholds for diagnosis of GDM at any time during pregnancy. Thus, GDM is diagnosed if any of the following diagnostic thresholds are met on the antenatal oral glucose tolerance test (OGTT): 0 h plasma glucose, 92–125 mg/dL or 5.1–6.9 mmol/L; 1 h plasma glucose, ≥180 mg/dL or 10 mmol/L; and 2 h plasma glucose, 153–199 mg/dL or 8.5–11.0 mmol/L. Normoglycemia is diagnosed when all the three plasma glucose values are below the diagnostic thresholds.

Given the high‐risk South Asian ethnicity, all women undergo universal screening for hyperglycemia during pregnancy. All women are advised a screening fasting plasma glucose (FPG) test at the first antenatal visit. Furthermore, 75 g OGTT and glycated hemoglobin (HbA1c) are advised on a case‐to‐case basis, especially including those with additional risk factors. Women with normoglycemia in early pregnancy (<20 weeks) are advised a repeat testing with one‐step 75 g OGTT at 24–28 weeks.

As per WHO 2013 criteria, ODiP was defined as FPG ≥126 mg/dL or 7 mmol/L and/or 2‐h post‐load plasma glucose ≥200 mg/dL or 11.1 mmol/L, at any time during pregnancy.^17^ The American Diabetes Association recommends that HbA1c is not reliable for screening of hyperglycemia beyond 15 weeks of gestation but can be used alongside other tests (FPG and 2‐h post‐load plasma glucose) for screening of undiagnosed diabetes in early pregnancy (<15 weeks).^18^ Therefore, we additionally used HbA1c ≥6.5% (48 mmol/mol), when available, to diagnose ODiP in women <15 weeks period of gestation. A single abnormal value for any of the three parameters was consistent with a diagnosis of ODiP. We did not use random plasma glucose for diagnosis of ODiP.

### 2.5 Antenatal OGTT data

Among women with normoglycemia and GDM (*n* = 826), complete OGTT data were available for 681 (82.4%) women (164 with normoglycemia and 517 with GDM). Data were not available for one or more OGTT values in the remaining 145 (17.6%) women. In such cases, the diagnosis of GDM or normoglycemia was ascertained from medical records and further corroborated by enquiring women during their study visits.

Missing OGTT data have implications for women with normoglycemia and GDM, as they could be reclassified into a higher category, but not for women with ODiP, who would not be reclassified. Therefore, to address this limitation, we performed a sensitivity analysis involving women with complete OGTT data, as detailed in the statistical analysis.

### 2.6 Primary outcome

Postpartum diabetes was defined as FPG ≥126 mg/dL or 7 mmol/L and/or 2‐h post load plasma glucose ≥200 mg/dL or 11.1 mmol/L and/or HbA1c ≥6.5% or 48 mmol/mol or receiving antihyperglycemic medications/previously diagnosed diabetes.^18^ Prediabetes was defined as FPG 100–125 mg/dL (5.6–6.9 mmol/L) and/or 2‐h post‐load plasma glucose 140–199 mg/dL (7.8–11.0 mmol/L) and/or HbA1c 5.7–6.4% (39–47 mmol/mol).^18^ Normoglycemia was defined as FPG <100 mg/dL (5.6 mmol/L), 2‐h post‐load plasma glucose <140 mg/dL (7.8 mmol/L), and HbA1c <5.7% (39 mmol/mol). HbA1c was not used for phenotyping prediabetes/diabetes in the participants (*n* = 5) tested in the immediate postpartum period (<12 weeks) due to its inherent inaccuracies in this time period.^13^

### 2.7 Secondary outcomes

Hypertension was defined as systolic blood pressure ≥140 mm Hg, and/or diastolic blood pressure ≥90 mm Hg on repeated examination (mean of three values taken 1 min apart) and/or the use of antihypertensive drugs.^19^ Overweight and obesity were defined as body mass index (BMI) ≥25 kg/m^2^ and ≥30 kg/m^2^, respectively.^20^ Metabolic syndrome was defined per International Diabetes Federation criteria, that is, waist circumference ≥80 cm plus two or more out of the following: serum triglycerides ≥150 mg/dL (1.7 mmol/L), FPG ≥100 mg/dL (5.6 mmol/L) or already on diabetes pharmacotherapy, high‐density lipoprotein cholesterol <50 mg/dL (1.29 mmol/L), and blood pressure ≥130/85 mm Hg or use of antihypertensive drugs.^21^ Insulin resistance was measured by the homeostatic model assessment of insulin resistance (HOMA‐IR) using the standard formula.^22^

### 2.8 Procedure on the day of testing

Eligible women were scheduled in a fasting state between 8:30 am and 9:00 am, and the first samples were obtained before 10 am in the majority. Women who were already on antihyperglycemic medications (*n* = 63 [27 and 36 in the GDM and ODiP group, respectively]) or were known cases of diabetes (*n* = 8 [3 and 5 in the GDM and ODiP group, respectively]) skipped OGTT and underwent FPG and HbA1c tests alone. All eight cases were confirmed to have diabetes on their present HbA1c result.

### 2.9 Sample collection and measurement

The 75 g OGTT involved collection of venous samples for plasma glucose at 0 and 2 h following the administration of a 75 g anhydrous glucose load. The women remained seated between the two samples, and during this time, the study staff filled the proforma capturing demographic, relevant antenatal, and medical details. Women also underwent weight, height, waist circumference, and blood pressure measurements using standardized methods.^23^ The biochemical samples like glucose, HbA1c/insulin, and lipid profile were obtained in gray top fluoride, purple top EDTA, and yellow top serum separator tubes, respectively. The details on sample transportation, processing, and analysis have been provided in one of our earlier publications.^23^

### 2.10 Sample size calculation

We calculated the sample size for our primary outcome, postpartum diabetes. Anticipating a postpartum diabetes prevalence of 10% in women with GDM (as reported by us earlier),^14^ and 25% in women with overt diabetes, and assuming an enrolment ratio of 6:1, significance level of 5% and power of 90%, we needed 426 women with GDM and 71 women with overt diabetes.

### 2.11 Statistical methods

We analyzed the data using Stata 15.0 (Stata Corp. LP, College Station, TX, USA) and presented it as number (%), mean (± SD) or median (q25‐q75) as appropriate. Qualitative variables were compared between the groups using the Pearson chi‐square test. Quantitative variables were assessed for normality using the Shapiro–Wilk test. Variables that followed normal distribution were compared using Student's *t* test for independent samples, and those that did not follow a normal distribution (triglycerides, duration since last follow‐up, pregnancy number, live births, HOMA‐IR) were compared using Wilcoxon rank‐sum test. To evaluate the factors associated with postpartum diabetes among women with ODiP, a logistic regression analysis was performed. The following factors were considered in the adjusted model: age (continuous), family history of diabetes and postpartum BMI (continuous) [Model 2], postpartum interval (continuous) [Model 3], history of GDM prior to the index pregnancy, pregnancy number (continuous) and another pregnancy after index case [Model 4], and education, and occupation [Model 5]. Model 1 was unadjusted and Model 6 was fully adjusted accounting for all the aforementioned covariates. The covariates selected for the analysis are well‐established risk factors for diabetes. Both crude and adjusted (95% confidence interval [CI]) odds ratios (ORs) were calculated. We also performed a sensitivity analysis and calculated the prevalence of postpartum diabetes among women with normoglycemia/GDM and complete antenatal OGTT data (ie, after excluding 145 [17.6%] women in the two groups with one or more missing OGTT values). Similarly, the adjusted ORs for postpartum diabetes in women with ODiP (ref: GDM) are also presented after accounting for the missing data in the latter group. A *p* value <.05 was considered statistically significant.

## 3 RESULTS

### 3.1 Baseline characteristics

We evaluated a total of 918 women for this study, with a mean ± SD age of 33.6 ± 5.0 years, BMI of 27.3 ± 5.2 kg/m^2^, and median (interquartile range [IQR]) postpartum interval of 31 (20–45) months. Women with ODiP were diagnosed at 18.4 ± 7.8 weeks period of gestation, GDM at 22.1 ± 6.1 weeks period of gestation, and normoglycemia at 23.7 ± 3.5 weeks period of gestation. Compared to women with GDM, those with ODiP were older, less likely to be educated (to graduation or beyond), were diagnosed earlier in the pregnancy, had a higher prepregnancy BMI and antenatal plasma glucose levels, and were more likely to have a family history of diabetes, history of GDM prior to the index pregnancy, and have used pharmacotherapy in their index pregnancy. There was no significant difference in the postpartum interval (29 [16–48] in ODiP vs. 33 [20–49] months in GDM; *p* = .223) between the two groups (Table 1). Among women who were >6 months postpartum at assessment (*n* = 886), 76% had a history of exclusive breastfeeding for 6 months.

**TABLE 1:** Baseline characteristics of study participants.

Variable	Total (*n* = 918)	NGiP (*n* = 183)	GDM (*n* = 643)	ODiP (*n* = 92)	*p* value (ODiP vs. GDM)
Age (years)	33.6 ± 5.0	31.3 ± 4.4	34.0 ± 5.0	35.2 ± 5.1	.023
Period of gestation at diagnosis#	22.1 ± 6.0	23.7 ± 3.5	22.1 ± 6.1	18.4 ± 7.8	<.001
Prepregnancy body mass index (kg/m^2^)^##^	25.2 ± 4.8	23.3 ± 4.3	25.4 ± 4.7	27.4 ± 5.1	< .001
Postpartum interval (months)	31 (20–45)	28 (21–37)	33 (20–49)	29 (16–48)	.223
Number of pregnancies	2 (2–4)	2 (1–3)	2 (2–4)	2 (2–3)	.374
Live births	2 (1–2)	1 (1–2)	2 (1–2)	2 (1–2)	.190
Education, graduate or above^	597 (65.1)	118 (64.5)	428 (66.7)	51 (55.4)	.034
Working status, employed^$^	202 (22.0)	26 (14.2)	154 (24.0)	22 (23.9)	.994
Past history of GDM/diabetes	94 (10.2)	1 (0.6)	69 (10.7)	24 (26.1)	<.001
Insulin prescribed	222 (30.2)	‐	152 (23.6)	70 (76.1)	<.001
Metformin prescribed	112 (15.2)	‐	85 (13.2)	27 (29.4)	<.001
Either of insulin or metformin	282 (38.4)	‐	205 (31.9)	77 (83.7)	<.001
Any pregnancy after index pregnancy	107 (11.7)	18 (9.8)	83 (12.9)	6 (6.5)	.078
Exclusive breastfeeding (in women who were >6 months postpartum at assessment)^^	673 (76.0)	130 (73.9)	475 (75.6)	68 (82.9)	.143
Family history of diabetes	417 (45.4)	54 (29.5)	304 (47.3)	59 (64.1)	.002
Plasma glucose 0 h (mmol/L)*	5.3 ± 1.1	4.4 ± 0.3	5.3 ± 0.6	7.1 ± 2.0	<.001
Plasma glucose 1 h (mmol/L)**	9.2 ± 2.5	7.2 ± 1.5	9.4 ± 2.1	12.9 ± 3.2	<.001
Plasma glucose 2 h (mmol/L)***	7.7 ± 2.4	6.2 ± 1.1	7.6 ± 1.8	12.4 ± 3.0	<.001

*Note*: Data expressed as number (%), mean ± SD, median (interquartile range), as appropriate.

Abbreviations: GDM, gestational diabetes; NGiP, normoglycemia in pregnancy; ODiP, overt diabetes in pregnancy.

^#^ *n* = 793

^##^ *n* = 736

^^^ *n* = 917

^^^^ *n* = 886

* *n* = 871

** *n* = 742

*** *n* = 798

^$^ Graduation or beyond corresponds to 15 years of formal education or more.

A total of 320 (34.9%) participants used contraception. Of these, 136 (42.5%) had intrauterine device insertion, 91 (28.4%) used barrier contraception, 78 (24.4%) underwent female sterilization, 10 (3.1%) were on oral contraceptive pills, and 5 (1.6%) used alternative means.

### 3.2 The prevalence of diabetes in the postpartum period

Postpartum diabetes was present in 65 (70.7%) women with ODiP compared to 99 (15.4%) women with GDM (*p* < .001) and 4 (2.2%) women with normoglycemia in pregnancy (*p* < .001) (Table 2). There were 33 participants without postpartum diabetes where the diagnosis was made based on 2/3 tests and one participant where the diagnosis was made based on 1/3 tests. Postpartum prediabetes and normoglycemia were present in 20 (21.7%) and 7 (7.6%) women with ODiP, 337 (52.4%) and 207 (32.2%) women with GDM, and 56 (30.6%) and 123 (67.2%) women with normoglycemia in pregnancy (Table 2).

**TABLE 2:** Distribution and prevalence of cardiometabolic risk factors among the study participants.

Variable	Total (*n* = 918)	NGiP (*n* = 183)	GDM (*n* = 643)	ODiP (*n* = 92)	*p* value (ODiP vs. GDM)
Fasting plasma glucose (mmol/L)*	6.1 ± 2.2	5.1 ± 0.6	5.9 ± 1.7	8.8 ± 4.1	<.001
HbA1c (%)**	5.9 ± 1.3	5.3 ± 0.5	5.8 ± 1.1	7.4 ± 2.2	<.001
HbA1c (mmol/mol)**	40.6 ± 14.2	34.3 ± 5.1	39.9 ± 12.1	57.9 ± 24.1	<.001
Normoglycemia	337 (36.7)	123 (67.2)	207 (32.2)	7 (7.6)	
Prediabetes	413 (45.0)	56 (30.6)	337 (52.4)	20 (21.7)	
Diabetes	168 (18.3)	4 (2.2)	99 (15.4)	65 (70.7)	<.001
Body mass index (kg/m^2^)	27.3 ± 5.2	25.5 ± 4.5	27.7 ± 5.3	27.8 ± 4.8	.861
Overweight/obese	610 (66.5)	95 (51.9)	451 (70.1)	64 (69.6)	.910
Waist circumference (cm)^	92.3 ± 11.1	88.0 ± 10.2	93.3 ± 11.2	94.1 ± 10.5	.499
Systolic blood pressure (mm Hg)***	113.4 ± 11.9	110.4 ± 11.0	113.5 ± 11.8	118.3 ± 12.8	<.001
Diastolic blood pressure (mm Hg)***	75.8 ± 9.0	74.3 ± 9.2	75.9 ± 9.1	77.8 ± 7.7	.052
Hypertension	64 (7.0)	8 (4.4)	49 (7.6)	7 (7.6)	.990
Total cholesterol (mmol/L)^^	4.4 ± 0.9	4.3 ± 0.9	4.4 ± 0.9	4.7 ± 0.9	<.001
Low‐density lipoprotein cholesterol (mmol/L)^^	2.4 ± 0.7	2.4 ± 0.7	2.4 ± 0.7	2.7 ± 0.8	<.001
High‐density lipoprotein cholesterol (mmol/L)^^	1.4 ± 0.3	1.4 ± 0.4	1.3 ± 0.3	1.3 ± 0.3	.836
Triglycerides (mmol/L)^^	1.1 (0.9–1.5)	1.0 (0.7–1.3)	1.2 (0.9–1.5)	1.4 (1.0–1.8)	.002
HOMA‐IR^^^	3.2 (2.1–4.8)	2.3 (1.5–3.3)	3.4 (2.3–5.0)	4.5 (3.0–7.0)	<.001
Metabolic syndrome	337 (37.2)	28 (15.4)	256 (40.4)	53 (58.2)	.001

Abbreviations: GDM, gestational diabetes; HbA1c, glycated hemoglobin; HOMA‐IR, homeostatic model assessment of insulin resistance; NGiP, normoglycemia in pregnancy; ODiP, overt diabetes in pregnancy.

* *n* = 915

** *n* = 911 (five women were less than 3 months postpartum)

*** *n* = 916

^^^ *n* = 917

^^^^ *n* = 912

^^^^^ *n* = 867

The prevalence of diabetes in ODiP group increased with the duration of follow‐up (47.4% [9/19] of all women tested in the first postpartum year, 65.7% [23/35] of all women tested at 1–3 years postpartum and 86.8% [33/38] of all women tested at >3 years postpartum). The corresponding figures for GDM group were 5.6% (5/90), 14.0% (39/278), and 20% (55/275), respectively. The unadjusted and adjusted ORs for diabetes among women with ODiP were 13.23 (95% CI 8.05, 21.75; *p* < .001) and 14.82 (95% CI, 8.49, 25.87; *p* < .001), respectively, considering GDM as a reference category (Table 3). Of 29 women with ODiP diagnosed in the first trimester, 27 (93.1%) developed postpartum diabetes, whereas of the 57 diagnosed in the second trimester, 35 (61.4%) developed postpartum diabetes (*p* = .005). Diabetes was more common when diagnosis of ODiP was based on antenatal FPG elevation (35/41, 85.4%) or HbA1c elevation before 15 weeks of gestation (19/20, 95%), compared to 2‐h post‐load plasma glucose elevation (33/56, 58.9%).

**TABLE 3:** Association between overt diabetes in pregnancy and postpartum diabetes (reference category: gestational diabetes mellitus).

Models	Covariates adjusted	Odds ratio	95% CI	*p* value
Model 1	Unadjusted	13.23	8.05–21.75	<.001
Model 2	Age, postpartum body mass index, family history of diabetes	13.92	8.26–23.46	<.001
Model 3	Duration since childbirth	15.17	9.05–25.43	<.001
Model 4	History of gestational diabetes in previous pregnancy other than the index one, pregnancy number, another pregnancy after index case	13.00	7.80–21.67	<.001
Model 5	Education and occupation	12.86	7.80–21.19	<.001
Model 6	All covariates adjusted in models 1 to 5	14.82	8.49–25.87	<.001

Abbreviation: CI, confidence interval.

On sensitivity analysis, performed after excluding 145 women with missing antenatal OGTT data, the prevalence of diabetes in the normoglycemia and GDM group was 2.4% (4/164) and 11.8% (61/517), respectively. The adjusted OR for diabetes among women with ODiP was 22.88 (95% CI 12.40, 42.22; *p* < .001) considering GDM (*n* = 517, complete OGTT data) as a reference category.

### 3.3 Factors associated with postpartum diabetes in women with ODiP

The risk of postpartum diabetes significantly increased with an increase in postpartum interval (OR 1.03 per month increase; 95% CI, 1.0008–1.06; *p* = .009). The factors that were associated with an overall effect size of >2.0 but not statistically significant were education to less than graduate levels (OR 2.45; 95% CI, 0.94–6.39; *p* = .067), and history of GDM prior to index pregnancy (OR 2.56; 95% CI, 0.78–8.36; *p* = .121) (Table 4).

**TABLE 4:** Factors associated with postpartum diabetes among women with overt diabetes in pregnancy.

Parameter	No diabetes	Diabetes	Bivariate odds ratio	95% CI	*p* value
Age	Continuous variable		1.09	0.99–1.21	.086
Previous history of gestational diabetes	4/27 (14.8)	20/65 (30.8)	2.56	0.78, 8.36	.121
Time since delivery (per month increase)	Continuous variable		1.03	1.0008–1.06	.009
Body mass index	Continuous variable		1.01	0.92–1.11	.902
Family history of diabetes	16/27 (59.3)	43/65 (66.2)	1.34	0.53, 3.38	.531
Education (less than graduation)	8/27 (29.6)	33/65 (50.8)	2.45	0.94, 6.39	.067
Unemployed	20/27 (74.1)	50/65 (76.9)	1.17	0.41, 3.29	.771
Exclusive breastfeeding (in women who were >6 months postpartum at assessment; *n* = 82)	18/21 (85.7)	50/61 (82.0)	1.32	0.33, 5.28	.695
Pregnancy number	Continuous variable		1.06	0.81–1.38	.694

Abbreviation: CI, confidence interval.

### 3.4 The distribution of cardiometabolic risk factors

#### 3.4.1 As continuous variables

Women with ODiP had significantly higher systolic blood pressure, total cholesterol, low‐density lipoprotein cholesterol, and triglycerides compared to their counterparts with GDM. However, there were no significant differences in body mass index, waist circumference, diastolic blood pressure, or high‐density lipoprotein cholesterol levels (Table 2). The median (IQR) HOMA‐IR was also significantly higher in women with ODiP compared to GDM (4.5 [3.0–7.0] vs. 3.4 [2.3–5.0]; *p* < .001).

#### 3.4.2 As categorical variables

The prevalence of metabolic syndrome was significantly higher in women with ODiP than in GDM (58.2 vs. 40.4%; *p* = .001). However, the prevalence of overweight/obesity (69.6 vs. 70.1%; *p* = .910) and hypertension (7.6 vs. 7.6%; *p* = .990) was not significantly different between the two groups (Table 2).

## 4 DISCUSSION

We evaluated the prevalence of postpartum diabetes and other cardiometabolic risk factors in women with ODiP and compared these with women with a history of GDM and normoglycemia in their index pregnancy. Postpartum diabetes was present in 70.7% women in ODiP group compared to 15.4% in GDM group and only 2.2% in the normoglycemia group. Postpartum diabetes was more common when ODiP was diagnosed in the first compared to the second trimester of pregnancy and when the diagnosis was based on antenatal fasting plasma glucose elevation or HbA1c elevation before 15 weeks of gestation compared to 2‐h post‐load plasma glucose elevation. Considering GDM as the reference group, the adjusted odds for diabetes in ODiP group was nearly 15‐fold. Women with ODiP also had significantly higher prevalence of metabolic syndrome and presented higher levels of systolic blood pressure and lipid parameters compared to women with GDM.

The data on development of postpartum diabetes following ODiP are limited and heterogeneous.^7^, ^8^, ^9^ The available studies have evaluated women close to the time of delivery and the diagnostic criteria for ODiP have been at variance with the standard guidance. For example, Wong et al evaluated 133 women with ODiP at 6–8 weeks postpartum and reported diabetes in 21% and prediabetes in 37.6%.^7^ Similarly, Park et al evaluated 71 women and found diabetes in 73% at 6–8 weeks postpartum.^8^ Both these studies defined ODiP based on a retrospective audit of women undergoing GTT between 24 and 28 weeks of gestation and thus missed out on ODiP in early pregnancy.^7^, ^8^ Similarly, Nabi et al prospectively evaluated 32 women and found diabetes in 14.8% (4/27) at 1 month and 25.0% (6/24) at 6 months postpartum.^9^ However, even this study excluded women diagnosed with hyperglycemia in pregnancy before 12 weeks of gestation. Thus, the patient population recruited in previous studies did not represent the complete spectrum of ODiP and raise the question of generalisability. We recruited women diagnosed with ODiP at all stages of pregnancy and found that the prevalence of postpartum diabetes was especially high (93.1%) in women diagnosed in the first trimester of pregnancy. A high prevalence of postpartum diabetes in women diagnosed at an early stage of pregnancy could also suggest that these women have unrecognized preexisting diabetes.

The risk of diabetes (adjusted OR) was nearly 15‐fold higher among women with ODiP in reference to those with GDM in the primary analysis and nearly 23‐fold higher in the sensitivity analysis (performed after excluding GDM women with incomplete antenatal OGTT data). Women with ODiP were more likely to have metabolic syndrome and had significantly higher systolic blood pressure, total cholesterol, low‐density lipoprotein cholesterol, triglycerides, and HOMA‐IR compared to their counterparts with GDM. There is ample evidence to suggest that GDM is a risk factor for future cardiovascular diseases.^10^ The data from our study suggest that the degree of association for future cardiovascular diseases could be much higher in women with ODiP. We found that nearly 50% of women with ODiP did not develop diabetes in the first year of follow‐up, and therefore, such women are amenable to prevention strategies. Furthermore, many women could still be at an early stage of diabetes and prolonged remission can be targeted; this postulation needs evaluation in future intervention studies. We also found that women with ODiP diagnosed in the first trimester of pregnancy and based on antenatal fasting plasma glucose elevation or HbA1c elevation before 15‐week period of gestation were more likely to have postpartum diabetes. These could serve as useful predictive factors in clinical practice. An increase in postpartum interval was significantly associated with an increased risk of diabetes in ODiP group. The overall OR was >2.0 for history of GDM and poor education (less than graduation level) as risk factors for postpartum diabetes among women with ODiP. However, the results lacked statistical significance perhaps due to relatively small number of women with ODiP in our study. We suggest that the utility of these factors for prediction and risk stratification should be evaluated in larger multicenter studies. From a clinical perspective, our study finding that a large proportion of women with ODiP develop postpartum diabetes suggests that (a) women should be emphatically sensitized during the pregnancy about the long‐term risks and consequences, and (b) intensive efforts for prevention of future diabetes should be initiated from the time of diagnosis and continued postpartum.

To the best of our knowledge, this is the first prospective study that evaluated long‐term postpartum glycemic and cardiometabolic outcomes among women with ODiP and GDM diagnosed using standard diagnostic criteria. Previous studies involved short‐term follow‐up and our study adds useful data on the prevalence of diabetes beyond 6 months postpartum. We acknowledge certain limitations. The number of women with ODiP was small (although higher than two of the three studies reported in the literature), limiting the scope for specific exploratory analysis. The study was conducted at a single tertiary care center in India, so the findings may not be reflective of rural, primary, or secondary care settings or for other ethnicities.

To conclude, the prevalence of diabetes was significantly higher in women with ODiP compared to GDM. Women diagnosed in the first trimester of pregnancy and based on antenatal fasting plasma glucose elevation or HbA1c elevation before 15 weeks of gestation were at higher risk for future diabetes. Early and intensive intervention can be aimed at nearly 50% of women who have not yet developed diabetes in the first year to prevent future diabetes. Intensive screening protocols need to be developed in the postpartum period for women with ODiP.

## AUTHOR CONTRIBUTIONS

Yashdeep Gupta: Conceptualized this project, executed it, did analysis, wrote the first draft of this manuscript, and edited the manuscript after incorporating feedback. Alpesh Goyal: Provided scientific inputs in analysis of data, helped in preparing the manuscript, edited the manuscript, and gave final approval. Samita Ambekar: Helped in execution of the project, helped in data analysis, edited the manuscript, and gave final approval. Mani Kalaivani: Helped in statistical analysis, edited the manuscript, and gave final approval. Neerja Bhatla: Helped in execution of the project, edited the manuscript, and gave final approval. Nikhil Tandon: Conceptualized this project, executed it, provided scientific inputs in preparing the protocol, edited the manuscript, and gave final approval.

## FUNDING INFORMATION

This project has been funded by Indian Council of Medical Research (Grant No: 55/4/8/CARE‐YD/2018‐NCD‐II). Nikhil Tandon is the primary recipient of this grant. The sponsor has no role in study design, data collection, analysis and interpretation, writing of the report, and the decision to submit the report for publication.

## CONFLICT OF INTEREST STATEMENT

Yashdeep Gupta, Alpesh Goyal, Samita Ambekar, Mani Kalaivani, Neerja Bhatla, and Nikhil Tandon have no disclosures to report.

## Data Availability

The data sets generated during and/or analyzed during the current study will be available from the corresponding author on reasonable request.

## References

[1] International Association of Diabetes and Pregnancy Study Groups Consensus Panel . International Association of Diabetes and Pregnancy Study Groups recommendations on the diagnosis and classification of hyperglycemia in pregnancy. Diabetes Care. 2010;33:676‐682. doi:10.2337/dc09-1848 20190296 PMC2827530

[2] McIntyre HD , Catalano P , Zhang C , Desoye G , Mathiesen ER , Damm P . Gestational diabetes mellitus. Nat Rev Dis Primers. 2019;5(1):47. doi:10.1038/s41572-019-0098-8 31296866

[3] International Diabetes Federation . IDF Diabetes Atlas—10th edition. Diabetes Atlas http://www.diabetesatlas.org/ 2021. 35914061

[4] Goyal A , Gupta Y , Tandon N . Overt diabetes in pregnancy. Diabetes Ther. 2022;13:589‐600. doi:10.1007/s13300-022-01210-6 35107789 PMC8991291

[5] de Graaff AA , Land JA , Kessels AG , Evers JL . Demographic age shift toward later conception results in an increased age in the subfertile population and an increased demand for medical care. Fertil Steril. 2011;95:61‐63. doi:10.1016/j.fertnstert.2010.05.013 20646685

[6] Andreu A , Casals G , Vinagre I , Flores L . Obesity management in women of reproductive age. Endocrinol Diabetes Nutr. 2023;70(Suppl 1):85‐94. doi:10.1016/j.endien.2022.11.015 36424339

[7] Wong T , Ross GP , Jalaludin BB , Flack JR . The clinical significance of overt diabetes in pregnancy. Diabet Med. 2013;30:468‐474. doi:10.1111/dme.12110 23278460

[8] Park S , Kim SH . Women with rigorously managed overt diabetes during pregnancy do not experience adverse infant outcomes but do remain at serious risk of postpartum diabetes. Endocr J. 2015;62:319‐327. doi:10.1507/endocrj.EJ14-0529 25735969

[9] Nabi T , Rafiq N , Arifa QA , Mishra S . Effect of overt diabetes and gestational diabetes mellitus on pregnancy outcomes and progression. J Obstet Gynaecol India. 2022;72(Suppl 1):235‐242. doi:10.1007/s13224-022-01649-4 35928066 PMC9343514

[10] Xie W , Wang Y , Xiao S , Qiu L , Yu Y , Zhang Z . Association of gestational diabetes mellitus with overall and type specific cardiovascular and cerebrovascular diseases: systematic review and meta‐analysis. BMJ. 2022;378:e070244. doi:10.1136/bmj-2022-070244 36130740 PMC9490552

[11] Diabetes in Pregnancy: Management from Preconception to the Postnatal Period. National Institute for Health and Care Excellence (UK); 2020. 32212588

[12] Diabetes Canada Clinical Practice Guidelines Expert Committee , Feig DS , Berger H , et al. Diabetes and pregnancy. Can J Diabetes. 2018;42(Suppl 1):S255‐S282. doi:10.1016/j.jcjd.2017.10.038 29650105

[13] ElSayed NA , Aleppo G , Aroda VR , et al. Management of diabetes in pregnancy: standards of care in diabetes‐2023. Diabetes Care. 2023;46(Suppl 1):S254‐S266. doi:10.2337/dc23-S015 36507645 PMC9810465

[14] Goyal A , Gupta Y , Kalaivani M , et al. Long term (>1 year) postpartum glucose tolerance status among Indian women with history of gestational Diabetes mellitus (GDM) diagnosed by IADPSG criteria. Diabetes Res Clin Pract. 2018;142:154‐161. doi:10.1016/j.diabres.2018.05.027 29802954

[15] Kubihal S , Gupta Y , Shalimar , et al. Prevalence of non‐alcoholic fatty liver disease and factors associated with it in Indian women with a history of gestational diabetes mellitus. J Diabetes Investig. 2021;12:877‐885. doi:10.1111/jdi.13411 PMC8089012 32961610

[16] Gupta Y , Goyal A , Kalaivani M , et al. High prevalence of cardiometabolic risk factors in spouses of Indian women with hyperglycemia in pregnancy. Diabet Med. 2020 Jun;37(6):1058‐1065. doi:10.1111/dme.14283 32112453

[17] Diagnostic criteria and classification of hyperglycaemia first detected in pregnancy: a World Health Organization guideline. Diabetes Res Clin Pract. 2014;103:341‐363. 24847517 10.1016/j.diabres.2013.10.012

[18] ElSayed NA , Aleppo G , Aroda VR , the American Diabetes Association , et al. 2. Classification and diagnosis of diabetes: standards of care in diabetes‐2023. Diabetes Care. 2023;46(Suppl 1):S19‐S40. doi:10.2337/dc23-S002 36507649 PMC9810477

[19] Unger T , Borghi C , Charchar F , et al. 2020 international society of hypertension global hypertension practice guidelines. J Hypertens. 2020;38:982‐1004. doi:10.1097/HJH.0000000000002453 32371787

[20] World Health Organisation . Overweight and obesity. 2018 Available from: www.who.int/news-room/fact-sheets/detail/obesity-and-overweight. Accessed 27 May 2023.

[21] International Diabetes Federation . The IDF consensus worldwide definition of the metabolic syndrome. 2006 Available from: www.idf.org/e‐library/consensus‐statements/60‐idfconsensus‐worldwide‐definitionof‐the‐metabolic‐syndrome. Accessed 27 May 2023.

[22] Matthews DR , Hosker JP , Rudenski AS , Naylor BA , Treacher DF , Turner RC . Homeostasis model assessment: insulin resistance and beta‐cell function from fasting plasma glucose and insulin concentrations in man. Diabetologia. 1985;28:412‐419. doi:10.1007/BF00280883 3899825

[23] Goyal A , Gupta Y , Kalaivani M , et al. Concordance of glycemic and cardiometabolic traits between Indian women with history of gestational diabetes mellitus and their spouses: an opportunity to target the household. Diabetologia. 2019;62:1357‐1365. doi:10.1007/s00125-019-4903-4 31104096

